# School and non‐school day screen time profiles and their differences in health and educational indicators in adolescents

**DOI:** 10.1111/sms.14214

**Published:** 2022-07-27

**Authors:** Pedro Antonio Sánchez‐Miguel, Javier Sevil‐Serrano, David Sánchez‐Oliva, Miguel Angel Tapia‐Serrano

**Affiliations:** ^1^ Department of Didactics of Musical, Plastic and Body Expression, Faculty of Teaching Training University of Extremadura Cáceres Spain; ^2^ Department of Didactics of Musical, Plastic and Body Expression, Faculty of Sports Sciences University of Extremadura Cáceres Spain

**Keywords:** academic achievement, fitness, health, sedentary, sedentary behavior

## Abstract

Sedentary behavior and screen‐based devices in particular have been negatively associated with a wide range of health and educational indicators. However, few have examined these relationships separately for school days and non‐school days, and none have used a person‐centered approach. This study aimed to identify school and non‐school day screen time profiles, as well as examine possible differences in health indicators (physical fitness, fatness, physical activity, sleep duration, and Mediterranean diet) and academic performance. This study involved the participation of 1573 Spanish adolescents aged 12–16 years (54.73% girls). Academic performance was measured through grades in Mathematics, Language, English, and Physical Education. Physical fitness was measured through a battery of tests (cardiorespiratory fitness was measured using the 20 m shuttle run test, and muscular strength with both handgrip and standing long jump tests), while fatness (skinfold thicknesses) was assessed with calipers. Finally, physical activity, screen time, sleep duration, and adherence to the Mediterranean diet were measured using self‐reported questionnaires. Hierarchical cluster analyses based on square Euclidian distances and Ward's method were performed based on daily minutes of screen time recorded on school and non‐school days. We identified four clusters labeled and described as: (1) “High‐high”: highest screen time on school and non‐school days; (2) “High‐low”: high screen time on school days and low screen time on non‐school days; (3) “Low‐high”: low screen time on school days and high screen time on non‐school days; (4) “Low‐low”: lowest screen time on school and non‐school days. Adolescents who belonged to the “High‐high” profile had worse health‐related behaviors (i.e., physical activity, sleep duration, and adherence to Mediterranean diet) and academic performance than most other profiles, while adolescents who belonged to “Low‐low” profile showed the opposite pattern. Adolescents in the “Low‐high” profile had a higher sleep duration on school days and better academic performance than those in the “High‐low” profile. No differences in body fat, cardiorespiratory fitness, and muscular strength were found between the four different profiles. The results suggest that adolescents who accumulated a large amount of screen time on school and non‐school days reported worse health‐related behaviors and academic performance. Moreover, adolescents who had high screen time on school days reported only a short sleep duration on school days and worse academic performance than on non‐school days. Conducting interventions to reduce screen time in these four profiles, particularly in the groups of students with more screen time on school days, becomes essential to improving adolescents' healthy lifestyles and academic performance.

## 1 INTRODUCTION

Sedentary behavior has been defined as time in any waking behavior characterized by energy expenditure ≤1.5 metabolic equivalents (METs), while in a sitting, reclining, or lying posture.^1^ A previous systematic review revealed that sedentary time ranges from 4 h and 18 min to 8 h and 26 min among European adolescents.^2^ This is worrying because a high amount of sedentary time has been related to negative health and educational indicators among adolescents.^3^

Within the time spent in sedentary behavior, screen time refers to the time spent on screen‐based devices such as televisions, computers, video games, or smartphones. A high amount of time spent on screen‐based behaviors has been related to negative consequences,^3^ such as low academic performance,^3^, ^4^ a higher prevalence of overweight/obesity,^5^ low physical fitness,^3^ and health‐risk behaviors (e.g., physical inactivity, insufficient sleep, and unhealthy diet).^6^, ^7^, ^8^ Given that a recent scoping review showed children and adolescents spent 3.6 h/day (1.3–7.9 h/day) on screen time and half of them did not meet screen time recommendations (i.e., ≤2 h/day), excessive screen time has become a growing public health concern worldwide.^9^ A recent study showed that Spanish adolescents spent approximately 6 h to screen‐based behaviors per day, and only 4% of them met screen time recommendations (≤2 h/day).^10^ Therefore, it seems important to analyze this health‐risk behavior at the adolescent stage because they already have access to numerous screen‐based activities.

Most studies have pointed out that adolescents spend more screen time on non‐school days than on school days,^10^, ^11^ but few have separately examined the relationship between screen time and a diverse range of health and educational indicators for school days and non‐school days. The majority showed that screen time on school days is more negatively related to academic performance,^12^, ^13^, ^14^ physical activity,^7^ sleep duration,^15^ or (un)healthy diet^16^ than on non‐school days. During non‐school days, these more maladaptive outcomes could be explained by the displacement hypothesis.^17^ Excessive screen time on school days could interfere with academic activities and affect the adoption of a healthy lifestyle because the day has only 24 h.^18^ However, as opposed to screen time on school days, screen time on non‐school days has been found to be more detrimental for body mass index^19^, ^20^ and body fat percentage.^21^ Therefore, although screen time appears to have more negative related on school days than non‐school ones, this seems to depend on the study variable.

The few existing studies that have investigated the relationships between screen time on school and/or non‐school days and different health and educational indicators adopted a variable‐centered approach.^22^ This approach is based on the premise that the relationship between variables is similar throughout the population studied. Correlations, regressions, and structural equation models are statistical analyses specific to this variable‐centered approach. However, adolescents could adopt different combinations of screen time on school and non‐school days. For example, they might spend a high amount of screen time on school and non‐school days or the opposite, as well as high screen time only on school days or only on non‐school days. Identifying these possible combinations based on screen time spent on school and non‐school days is imperative to understand possible differences in health and academic indicators or how they should be addressed in interventions.

To best of our knowledge, no studies have examined screen time profiles using total daily minutes of screen time on school and non‐school days to perform a cluster analysis. This person‐centered approach makes it possible to classify individuals into homogeneous groups whose members have similar characteristics (e.g., classify students into different screen time groups according to time spent on school and non‐school days). The only existing study examined screen time profiles on school and non‐school days separately and, consequently, the total daily minutes of screen time on school and non‐school days were not combined.^23^

Therefore, relying on a person‐centered approach, the first aim was to identify screen time profiles using total daily minutes of screen time on school and non‐school days among a sample of Spanish adolescents. The second aim was to examine differences in health‐related behaviors (physical activity, sleep, and adherence to the Mediterranean diet), physical fitness, fatness, and academic performance according to the resulting screen time profiles.

## 2 MATERIALS & METHODS

### 2.1 Participants

This cross‐sectional study is part of the project “Promoting Healthy Lifestyles” conducted among Spanish adolescents.^24^ All students in the first and second years of secondary education from 22 high schools located in southwestern Spain (omitted for a blind review) were invited to participate. The study was conducted between March and June 2019, before the COVID‐19 pandemic. During this period, baseline assessments were completed in different high schools. From an initial sample of 2217 participants, 644 were excluded for the following reasons: 614 did not report screen time, 4 did not report physical activity, and 26 did not report sleep duration. A final sample of 1573 adolescents, aged 12–16 years old (861 girls and 712 boys), participated in this study (the student response rate was 70.95%).

The adolescents and their parents or guardians were informed about the aims of this study, and all provided written informed consent for participation. This study was approved by the Declaration of Helsinki and Ethics Committee of the University of Extremadura (89/2016).

### 2.2 Measures

#### 2.2.1 Sociodemographic factors

Participants self‐reported age and sex, while socioeconomic status was obtained according to the average income level per household unit in each of the cities/towns of the region of Extremadura.^25^

#### 2.2.2 Health‐related behaviors

##### Screen time

Screen‐based behaviors of the Youth Leisure‐Time Sedentary Behaviour Questionnaire (YLSBQ) were assessed.^26^ The YLSBQ is a valid and reliable questionnaire to assess screen time among Spanish children and adolescents.^26^ The questionnaire assesses the amount of screen time spent during both school and non‐school days (e.g., during the last week, how much time did you usually spend watching television on school days/on non‐school days?). Adolescents self‐reported the habitual time spent on television, video games, computers, and smartphones separately on both school and non‐school days. Seven possible answers were available for each screen‐based device: no time, 30 min, 1, 2, 3, 4, and ≥5 h. Usual screen time on school and non‐school days was measured by the sum of time spent on each screen device on school and non‐school days, respectively (usual television, video gaming, computer, and smartphone duration on school days and non‐school days).

##### Physical activity

Physical activity was assessed using the Spanish version^27^ of the Physical Activity Questionnaire for Adolescents (PAQ‐A).^28^ The PAQ‐A is valid and reliable for measuring physical activity levels in Spanish adolescents.^27^ This instrument has nine questions that measure the frequency of participation in physical activities in the previous 7 days at different moments, including during physical education, school breaks, lunchtime, after school, evenings, and weekends. Each item is scored on a 5‐point Likert scale ranging from 1 (low physical activity) to 5 (high physical activity). Once a value from 1 to 5 is obtained for each of the 9 items, the average value of these 9 items is taken, resulting in the final physical activity index.

##### Sleep duration

Sleep duration was assessed using a Spanish translation of a valid and reliable sleep questionnaire.^29^ Adolescents self‐reported their typical bedtime and wake‐up time on school and non‐school days in the preceding 7 days. These questions have been shown to be a valid (*r* = 0.45–0.90) and reliable (ICC = 0.71–0.99) measure for examining sleep duration in children aged 9–12 years.^30^

##### Adherence to the Mediterranean diet

This diet has been characterized by a high quantity of vegetables, legumes, cereals, fish, fruits, nuts, edible grains and bread, potatoes, poultry, beans, and olive oil, as well as being low in red meat.^31^ The Mediterranean diet has been positively associated with a wide range of health indicators such as health‐related quality of life and cardiorespiratory and muscular fitness.^31^, ^32^, ^33^, ^34^ Adherence to the Mediterranean diet was assessed by the Spanish updated version of the Mediterranean Diet Quality Index for Children and Adolescents (KIDMED).^35^, ^36^ This questionnaire has proven to be valid and reliable in Spanish youth.^35^ The KIDMED consists of 16 yes/no questions about food consumption, of which 12 questions are positively scored (+1, e.g., consumption of fresh or cooked vegetables more than once a day) and four are negatively scored (−1, e.g., going to a fast‐food restaurant more than once a week). Consistent with previous studies, the adherence to the Mediterranean diet index is calculated as the sum of each answer and ranges from −4 to 12.

#### 2.2.3 Physical fitness

Cardiorespiratory fitness and muscular strength were included as a measure of physical fitness, since a systematic review conducted by Carson et al.^3^ reported that both were most consistently associated with screen‐based behaviors.

##### Cardiorespiratory fitness

Cardiorespiratory fitness was measured using the 20 m shuttle run test by a member of the research team at the school. Participants were instructed to run between two lines separated by 20 m at a cadence set from a pre‐recorded audio file. The frequency of the sound signals progressively increased at each level (1 min per level) by 0.5 km/h from an initial speed of 8.5 km/h. The test ended when students failed to reach the end line at the prescribed pace on three consecutive attempts or voluntarily discontinued the test. The final score, computed as the number of stages completed, were converted into ml/kg/min using the equation of Leger et al.^37^

##### Muscular strength

Muscular strength was assessed using both the handgrip and standing long jump tests.^38^ A hand‐held dynamometer, with an adjustable grip (TKK 5101 Grip D; Takey), was used to measure upper body strength. The test was complete twice and the maximum score in kilograms for each hand was recorded. The average score of the left and right hand was calculated. To account for differences in body size, handgrip strength was expressed per kilogram of body weight. The standing long jump test was used to assess lower limb strength. Participants stood behind the starting line, with feet together, and jumped forward as far as possible. The distance was measured from the starting line to the point where the back of the heel nearest to the take‐off line landed on the ground. Participants completed the test twice and the longest distance was recorded in centimeters. According to previous studies,^39^, ^40^, ^41^ muscle strength was calculated by adding the *z*‐score (upper and lower body strength) and dividing by 2 to average them.

#### 2.2.4 Fatness

Fatness measurements were performed by two members of the research team of the same sex as the students being evaluated. These persons were specialized in skinfold measurements (the highest official certificate in Spain), in order to reduce possible measurement errors. The measurements were carried out at school, in a room close to their usual classroom.

##### Body fat percentage

Skinfold thicknesses were measured on the non‐dominant side of the body to the nearest 0.1 mm with a Holtain caliper on the triceps and subscapular sites. The body fat percentage was calculated from the triceps and subscapular skinfold thicknesses using Slaughter's equations.^42^ Two non‐consecutive measurements were performed on the non‐dominant side of the body and averages were recorded.^38^ Skinfold thicknesses were also assessed twice, and averages were recorded. Both measurements were conducted with participants dressed in shorts and a T‐shirt.

#### 2.2.5 Academic performance

##### Academic performance

School grades for four subjects: First Language (Spanish), Mathematics, Foreign Language (English), and Physical Education were provided at the end of the academic year by each high school. Consistent with previous studies,^43^, ^44^ academic performance was calculated as an average of First Language (Spanish), Mathematics, Foreign Language (English), and Physical Education grades. The average rating score can range from 0 to 10 in each subject.

### 2.3 Data analysis

Data analyses were performed using JASP software version 0.13.12020 (JASP Team University of Amsterdam, Netherlands). We adopt an alpha level of 5% for statistical significance.

#### 2.3.1 Descriptive and correlational analyses

The mean, standard deviation (SD), or percentage (%) was calculated for the sociodemographic characteristics, health‐related behaviors, body fat, physical fitness, and academic performance. The distribution of all variables was checked both by means of graphs (normal probability plots) and statistics (Kolmogorov–Smirnov test; *p* > 0.05). Differences between boys and girls were tested using Student's *t*‐test for continuous variables and chi‐square test for categorical variables. Pearson bivariate correlations between all study variables were conducted (i.e., higher Pearson correlation coefficients represent a greater degree of relationship).

#### 2.3.2 Cluster analysis

Cluster analysis was conducted based on the standardized scores of total daily minutes of screen time on school and non‐school days. Cluster analysis was divided into two steps.^45^ First, a hierarchical cluster analysis was conducted using Ward's method based on squared Euclidean distances.^46^, ^47^ Second, k‐means cluster analysis was used to obtain the final cluster solution, including the initial cluster centers extracted for each possible number of cluster solutions from the first step. The optimal number of profiles was determined through the examination of statistical indicators (Bayesian Information Criterion [BIC] and Akaike Information Criteria [AIC]), the statistical adequacy of the solution (i.e., lower scores in BIC and AIC are better), and the substantive meaning and theoretical conformity of the extracted profile (i.e., the solution is theoretically reasonable and parsimonious and there are no clusters with <10% of the participants). To examine the reliability and stability of the final cluster solution, the sample was randomly split into two halves, and the same two steps previously described (Ward's and K‐means) were tested.^48^ Cohen's kappa coefficient was used to assess the degree of agreement between the classification of adolescents of each of the new subsamples and the original sample. An agreement of at least 0.60 is considered acceptable.^46^

#### 2.3.3 School and non‐school day screen time profiles and their differences in health and educational indicators

Chi‐square and Cramer's *V* tests were used to determine the association of cluster solutions with sex. Cramer's *V* values above 0.10 were considered small, above 0.30 medium, and above 0.50 large.^49^ Cluster differences in health‐related behaviors (i.e., physical activity, Mediterranean diet, and sleep duration), physical fitness (i.e., cardiorespiratory fitness and muscular strength), fatness, and academic performance were conducted using a covariance analysis (ANCOVA). Since significant differences were found between screen time profiles and age, sex, and socioeconomic status, they were added as covariates or confounding variables. Physical activity and Mediterranean diet were also added as covariates when they were not used as dependent variables. Tukey's post hoc test was conducted when significant differences were found in the study variables between the different profiles. Effect sizes above 0.01 were considered small, >0.06 moderate, and over 0.14 large.^49^

## 3 RESULTS

### 3.1 Descriptive and correlational analyses

Descriptive statistics and prevalence of the different health and educational indicators by sex are displayed in Table 1. Girls showed significantly higher scores in sleep duration on non‐school days, body fat percentage, and academic performance compared with boys (all, *p* < 0.01), whereas boys reported significantly higher values in screen time on school and non‐school days, physical activity, cardiorespiratory fitness, and muscular strength compared to girls (all, *p* < 0.001). As displayed in File S1, screen time spent on school and non‐school days were negatively related to physical activity (*r* = −0.11 and *r* = −0.08, respectively), sleep duration on school (*r* = −0.31 and *r* = −0.09, respectively) and non‐school days (*r* = −0.12 and *r* = −0.31, respectively), adherence to the Mediterranean diet (*r* = −0.24 and *r* = −0.17, respectively), and academic performance (*r* = −0.22 and *r* = −0.10, respectively) and positively related with muscular strength (*r* = 0.09 and *r* = 0.07, respectively). A low negative relationship between screen time on school days and cardiorespiratory fitness was also found (*r* = −0.07). The degree of relationship was higher between the different study variables and screen time on school days compared with screen time on non‐school days, except for sleep duration on non‐school days. For more detailed information, correlation analyses have been added in File S1.

**TABLE 1:** Descriptive statistics and prevalence of the different health and academic indicators by sex

Measurements	Total sample *n* = 1573	Girls *n* = 712	Boys *n* = 861	*p*‐Value
	*M ±* SD	*M ±* SD	*M ±* SD	
Sociodemographic characteristics				
Age (years)	13.05 *±* 0.86	12.98 *±* 0.82	13.10 *±* 0.88	<0.01
Socioeconomic status (€)	21238.25 *±* 3018.85	21249.59 *±* 3098.29	21229.32 *±* 2957.14	0.910
Health‐related behaviors				
Screen time on school days (min/day)	165.31 *±* 127.23	152.76 *±* 123.67	171.62 *±* 131.08	<0.01
Screen time of non‐school days (min/day)	365.55 *±* 201.95	302.69 *±* 173.27	411.68 *±* 208.63	<0.001
Physical activity (1–5)	3.10 *±* 0.75	2.92 *±* 0.73	3.25 *±* 0.73	<0.001
Sleep duration on school days (h/day)	8.27 *±* 0.99	8.24 *±* 0.89	8.343 *±* 0.98	0.070
Sleep duration on non‐school days (h/day)	9.52 *±* 1.64	9.81 *±* 1.45	9.40 *±* 1.73	<0.001
Mediterranean diet (−4 to 12)	5.78 *±* 2.49	5.73 *±* 2.57	5.81 *±* 2.42	0.619
Body fat (%)	27.84 *±* 10.76	28.90 *±* 7.79	27.00 *±* 12.57	<0.001
Physical fitness				
Cardiorespiratory fitness (ml/kg/min)	42.52 *±* 5.95	39.94 *±* 4.30	44.55 *±* 6.27	<0.001
Standing long jump (cm)	158.59 *±* 44.08	142.41 *±* 27.09	171.32 *±* 50.28	<0.001
Handgrip strength/weight	0.47 *±* 0.05	0.44 *±* 0.11	0.48 *±* 0.11	<0.001
Muscular strength (*z*‐score)	0.00 *±* 0.88	−0.31 *±* 0.70	0.24 *±* 0.93	<0.001
Academic performance (0–10)	7.20 *±* 1.65	7.36 *±* 1.61	7.07 *±* 1.68	<0.01

*Note*: Muscular strength: handgrip + standing long jump.

Abbreviations: *M*, medium; SD, standard deviation.

### 3.2 Cluster analysis

**FIGURE 1:**
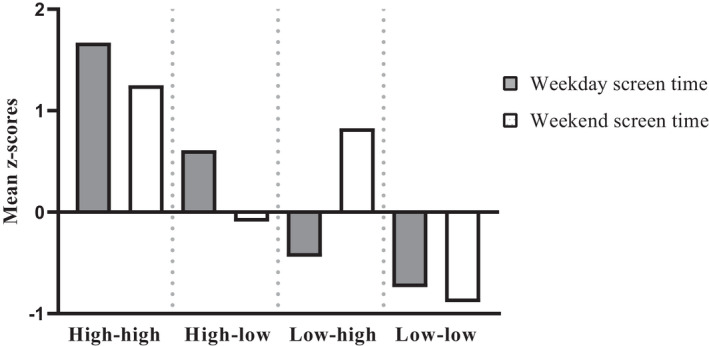
Four‐cluster solution based on *z*‐scores for both screen time on school and non‐school days

We estimated models for 2–10 profiles. The four‐cluster solution was selected as the best representation, based on the visual inspection of AIC and BIC and on theoretical assumptions. Regarding the BIC and AIC, we found a decrease on the slope in the four/five cluster solution; see File S2. Furthermore, when moving from four to five clusters, we found two clusters with non‐substantial differences in time spent on screen‐based devices. In addition, the number of participants in one of the groups was lower than 10%. This four‐cluster solution also showed good stability and replicability (*K* = 0.83). Figure 1 shows the graphical results for the four‐cluster solution based on *z*‐scores for school and non‐school screen time. The *z*‐score and raw score values for the four profiles are also presented in Table 2. All profiles were significantly different from each other in terms of screen time on school and non‐school days (*p* < 0.05). Cluster 1, labeled “High‐high”, included 15.2% of the sample (*n* = 239), and was characterized by a high amount of time spent on screen‐based behaviors on school and non‐school days. Cluster 2, “High‐low”, represented 22.6% of the sample (*n* = 356), and was characterized by high screen time on school days and low screen time on non‐school days. Cluster 3, “Low‐high”, contained 22.3% of the sample (*n* = 351), and was represented by students with low screen time on school days and high screen time on non‐school days. Cluster 4, “Low‐low”, represented 39.9% of the sample (*n* = 627), included adolescents with low screen time on school and non‐school days. The chi‐square test revealed a significant cluster assignment by sex (*χ* ^2^ = 91.64, *p* < 0.001). Clusters 1 and 3 had fewer girls than boys (32.22% vs. 67.78% and 28.49% vs. 71.51%, respectively), whereas clusters 2 and 4, although with smaller differences, had more girls than boys (51.40% vs. 45.60% and 56.14% vs. 43.86%, respectively).

**TABLE 2:** Associations of the four‐cluster solution and different health and educational indicators

Profiles	Cluster 1: High‐high	Cluster 2: High‐low	Cluster 3: Low‐high	Cluster 4: Low‐low	*χ* ^2^	*p*‐Value	Cramer's *V*
*n* (%)	239 (15.2)	356 (22.6)	351 (22.3)	627 (39.9)			
Sex							
Girls *n* (%)	77 (32.22)	182 (51.40)	100 (28.49)	352 (56.14)	91.64	<0.001	0.241
Boys *n* (%)	162 (67.78)	174 (45.60)	251 (71.51)	275 (43.86)			

	High‐high	High‐low	Low‐high	Low‐low	*F*	*p*‐Value	*ηp* ^2^
	*M* ± SD	*M* ± SD	*M* ± SD	*M* ± SD			
School and non‐school screen time profiles							
Screen time on school days (*z*‐scores)	378.26 (1.67)_bcd_	244.08 (0.61)_acd_	112.78 (−0.44)_abd_	77.17 (−0.74)_abc_	1239.59	<0.001	0.753
Screen time of non‐school days (*z*‐scores)	610.90 (1.25)_bcd_	346.69 (−0.10)_acd_	524.99 (0.83)_abd_	189.58 (−0.89)_abc_	1054.50	<0.001	0.671
Health‐related behaviors							
Physical activity	2.93 ± 0.05_d_	3.07 ± 0.04	3.02 ± 0.04_d_	3.16 ± 0.03_ac_	6.05	<0.001	0.011
Sleep duration on school days	7.81 ± 0.06_bcd_	8.14 ± 0.05_acd_	8.41 ± 0.05_ab_	8.43 ± 0.04_ab_	27.92	<0.001	0.050
Sleep duration on non‐school days	8.83 ± 0.11_bcd_	9.73 ± 0.08_a_	9.09 ± 0.09_ad_	9.94 ± 0.07_ac_	36.45	<0.001	0.066
Mediterranean diet	4.98 ± 0.16_cd_	5.47 ± 0.13_d_	5.58 ± 0.13_ad_	6.23 ± 0.10_abc_	17.93	<0.001	0.032
Body fat (%)	28.99 ± 0.72	28.26 ± 0.57	29.07 ± 0.59	28.32 ± 0.44	0.53	0.659	0.001
Physical fitness							
Cardiorespiratory fitness	41.53 ± 0.40	42.13 ± 0.32	42.07 ± 0.33	42.53 ± 0.25	1.48	0.217	0.003
Muscular strength	0.06 ± 0.07	0.06 ± 0.05	−0.13 ± 0.06	−0.07 ± 0.04	2.93	0.033	0.007
Academic performance	6.85 ± 0.10_cd_	7.06 ± 0.08_c_	7.37 ± 0.08_ab_	7.26 ± 0.06_a_	6.56	<0.001	0.011

^a^ *p* <0.05 vs. Cluster 1; ^b^ *p* <0.05 vs. Cluster 2; ^c^ *p* <0.05 vs. Cluster 3; ^d^ *p* <0.05 vs. Cluster 4.

### 3.3 School and non‐school day screen time profiles and their differences in health and educational indicators

**FIGURE 2:**
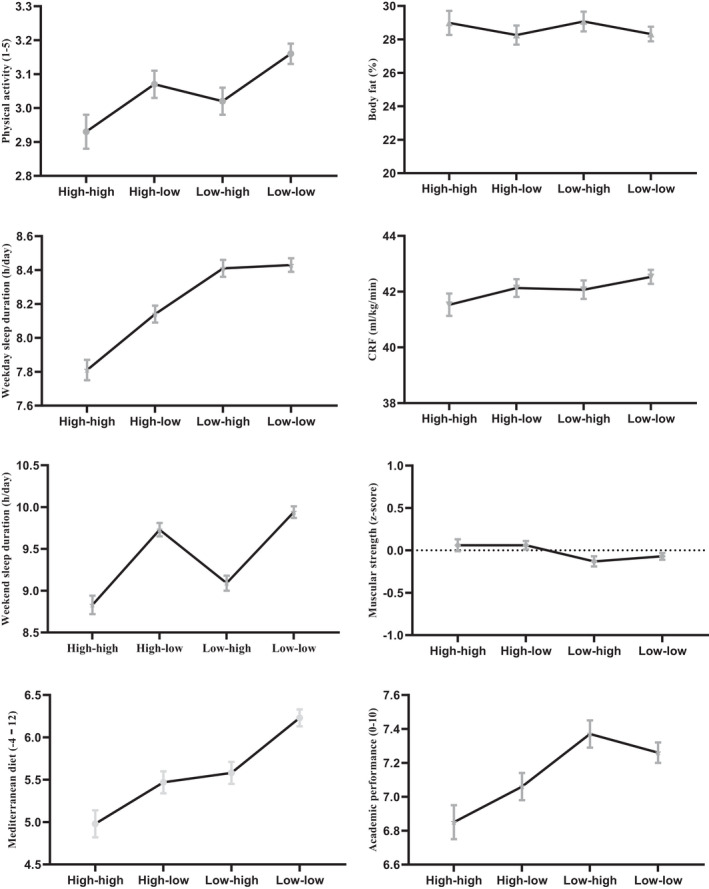
Four‐cluster solution based on school and non‐school day screen time

Pairwise comparisons between the four‐cluster solution, univariate *F*‐values, and effect sizes (*ηp* ^2^) are reported in Table 2. For the main effects, ANCOVAs revealed significant differences in physical activity (*F*_DF = 3_ = 6.05, *p* < 0.001), sleep duration on school days (*F*_DF = 3_ = 27.92, *p* < 0.001) and on non‐school days (*F*_DF = 3_ = 36.45, *p* < 0.001), adherence to the Mediterranean diet (*F*_DF = 3_ = 17.93, *p* < 0.001), as well as muscular strength (*F*_DF = 3_ = 2.93, *p* < 0.05), and academic performance (*F*_DF = 3_ = 6.56, *p* < 0.001) according to cluster membership. No significant differences were found between the four profiles in body fat percentage and cardiorespiratory fitness (all, *p* > 0.05). Specifically, the main differences between the four‐cluster solution and different health and educational indicators are summarized below (Figure 2).

Cluster 1: “High‐high”. These adolescents had lower physical activity levels compared to cluster 4. Moreover, adolescents from cluster 1 showed lower sleep duration on school and non‐school days compared with adolescents from clusters 2, 3, and 4. Adolescents who belonged to cluster 1 had a lower Mediterranean diet index and academic performance than clusters 3 and 4. Body fat, cardiorespiratory fitness, and muscle strength showed no significant differences in adolescents of the four profiles (*p* > 0.05).

Cluster 2: “High‐low”. Adolescents belonging to cluster 2 did not show significant differences on physical activity levels when compared with adolescents from clusters 1, 3, and 4. According to the sleep duration on school days, cluster 2 revealed a longer sleep duration than cluster 1, and a shorter one compared with clusters 3 and 4. Adolescents belonging to cluster 2 also reported a longer sleep duration during non‐school days than adolescents from cluster 1. In addition, these participants had a poor Mediterranean diet adherence and lower academic performance compared to adolescents from clusters 3 and 4, respectively.

Cluster 3: “Low‐high”. Adolescents belonging to cluster 3 showed a higher level of physical activity than adolescents from cluster 4, whereas adolescents belonging to cluster 3 did not report significant differences in physical activity levels when compared to adolescents from clusters 1 and 2. These adolescents revealed a higher sleep duration on school days compared with adolescents from clusters 1 and 2. However, adolescents belonging to cluster 3 had a lower sleep duration on non‐school days compared with adolescents from clusters 1 and 2. Adolescents in cluster 3 reported higher scores for the Mediterranean diet than adolescents from cluster 1, but poorer than cluster 4. Finally, adolescents from cluster 3 obtained a higher academic performance than adolescents in clusters 1 and 2.

Cluster 4: “Low‐low”. These participants reported higher physical activity scores and higher sleep duration on non‐school days in comparison with clusters 1 and 3. In addition, students included in this group revealed a higher sleep duration on school days compared with cluster 1 and 2. Moreover, these adolescents reported a better adherence to the Mediterranean diet compared with other profiles. Finally, students from cluster 4 reported higher academic performance in comparison with cluster 1.

## 4 DISCUSSION

The main findings of the study revealed that (1) four combinations of screen time on school and non‐school days were identified in this sample of adolescents (“High‐high”, “High‐low”, “Low‐high”, and “Low‐low” profiles), (2) adolescents with the highest scores in screen time on school and non‐school days displayed the worst health and educational indicators, while adolescents with the lowest scores in screen time on school and non‐school days showed the opposite pattern, (3) screen time on school and non‐school days can be negatively related to the adoption of a healthy lifestyle and the achievement of good grades, but not for body fat (%), cardiorespiratory fitness, and muscular strength, and (4) high screen time on school days only appears to be more damaging to sleep duration on school days as well as to academic performance.

Firstly, this study aimed to identify screen time profiles using total daily minutes of screen time on school and non‐school days. Analyzing school and non‐school day screen time profiles is necessary not only to identify possible combinations, but also to examine their implications for adolescent health. Relying on a person‐centered approach, four combinations of screen time on school and non‐school days were identified in this sample of Spanish adolescents. A profile characterized by low screen time on school and non‐school days (“Low‐low”) was composed of almost 40% of adolescents. Moreover, another profile of adolescents with the opposite combination (“High‐high”) was found, accounting for 15% of the sample. However, these two profiles only represent 55% of the adolescents of this study. The other two profiles characterized by low high screen time on school days and high screen time on non‐school days (“Low‐high”) and the opposite (“High‐low”) found account for 22.3% and 22.6% of the sample, respectively. Although in the “High‐low” and “Low‐low” profiles, there were approximately the same number of boys and girls, in the “Low‐high” and “High‐high” profiles, there were more boys. This could be because boys typically spend more time on screens, especially on non‐school days, while girls spend more time on other more educational or social sedentary behaviors.^11^, ^50^ All these cluster results suggest that, although approximately half of the adolescents have the same amount of time spent on school as on non‐school days (“Low‐low” or “High‐high”), the other half of the adolescents spend more screen time during school or non‐school days (“High‐low” or “Low‐high”). It is important to note that in absolute values, screen time on school days was higher than screen time on non‐school days in the four profiles identified in this study. The labeling of the profiles is thus a matter of gradation. In fact, none of the profiles met non‐school screen time recommendations of 120 min/day,^10^, ^11^ while the “Low‐high” and “Low‐low” profiles met screen time recommendations for school days. Most studies have also pointed out that adolescents spend more screen time on non‐school days than on school days, as well as that boys spend more time on screen‐based devices.^10^, ^11^ Because this is the first person‐centered study that combined screen time spent on school and non‐school days to perform a cluster analysis it was not possible to compare it with previous studies.

Having identified different combinations of school and non‐school screen time profiles, the second objective was to extend previous research by examining their possible differences in health‐related behaviors (physical activity, sleep, and Mediterranean diet), fitness, fatness, and academic performance. In relation to health‐related behaviors, the results of the present research showed significant differences between the four identified profiles and physical activity, sleep duration, and adherence to the Mediterranean diet. Adolescents belonging to the “High‐high” profile reported worse health‐related behaviors, while adolescents from the “Low‐low” profile showed higher values in health‐related behaviors. However, there were no significant differences in physical activity, Mediterranean diet adherence, and sleep duration on non‐school days between the “High‐low” and “Low‐high” profiles, except for sleep duration on school days, which was higher in the “Low‐high” profile. These results suggest that adolescents with high values for screen time on both school and non‐school days reported worse physical activity, sleep duration, and Mediterranean diet. In particular, adolescents with high screen time on school days reported a shorter sleep duration on school days.

The majority of previous variable‐centered studies have also shown a negative relationship between high screen time and health‐related behaviors such as physical activity^6^, ^7^, ^8^ and sleep duration.^6^, ^7^, ^8^ The negative outcomes associated with screen time for physical activity and sleep duration may be explained by the time displacement hypothesis.^17^ Excessive screen time could interfere with the adoption of higher levels of physical activity and an optimal sleep duration because these three (non)movement behaviors interact throughout the 24‐h period.^18^ A previous systematic review found an inverse association between sedentary behaviors and physical activity, supporting the displacement hypothesis.^6^, ^7^, ^8^ In addition, late‐night exposure to screens could be another mechanism that helps explain this negative relationship between screen time and sleep duration. Although our research did not measure the use of screen‐based devices at night, most previous studies indicate that adolescents spent a high proportion of time using screens at bedtime.^51^ According to a previous systematic review and a meta‐analysis, the short‐wavelength (blue/green) light emitted by screen‐based devices suppresses pineal melatonin secretion, influencing both circadian timing and sleep onset.^52^, ^53^ Therefore, late‐night screen time can lead to sleep disturbances,^54^ as well as delayed sleep onset.^55^ In addition, the content, its interactivity, and subsequent level of arousal that certain screen media have (e.g., video games) may also be negatively related to sleep time and quality.^56^

Consistent with the findings of a previous systematic review of variable‐centered studies,^6^, ^7^, ^8^ significant differences between the four identified profiles and the Mediterranean diet were found in our study. Participants who belong to the “Low‐low” profile showed a higher adherence to the Mediterranean diet than other profiles in the present study. In particular, other studies have suggested that the use of recreational screen time such as video games or television is often associated with the consumption of ultra‐processed foods in adolescents. This could be because when adolescents spend time engaging in screen‐based devices, they are not aware of their eating habits.^57^ The lower Mediterranean diet adherence found in the “High‐high” profile in our study could also be explained by the fact that adolescents in this profile also reported shorter sleep duration and lower levels of physical activity. Therefore, in line with a previous systematic review, a lower compliance with 24‐h Movement Guidelines could also explain a poor Mediterranean diet.^6^, ^7^, ^8^, ^58^, ^59^

Contrary to most previous variable‐centered studies that have shown how a high amount of screen time on school days can be more detrimental to health‐related behaviors such as physical activity,^7^ sleep duration,^15^ and healthy diet,^16^ in this person‐centered study only sleep duration on school days was lower in the profiles of adolescents with high screen time on school days. The displacement hypothesis could also explain why sleep duration during school days is shorter in those adolescents who spend more time on screen‐based devices during school days. Although significantly lower sleep time values on non‐school days were found in the “Low‐high” profile compared to the “Low‐low” profile, no differences were observed between the “High‐low” and “Low‐high” profiles. These results suggest that since adolescents do not have to go to school on non‐school days, high screen time on non‐school days does not seem to be related to sleep duration on non‐school days, since they may wake up later. However, consistent with aforementioned previous studies,^7^, ^15^, ^16^ it should be noted that the correlation analysis identified a higher degree of relationship between screen time on school days and health‐related behaviors in this study. Further studies using a person‐centered approach are required to shed light on whether screen time on school and non‐school days can be more harmful in health‐related behaviors.

With respect to body fat and physical fitness (i.e., muscular strength and cardiorespiratory fitness), there were no significant differences among the four clusters identified in this research. Previous systematic reviews have found that the use of some recreational screen‐based behaviors such as video games and/or computers appears to have a detrimental consequences on body composition^60^ and physical fitness.^3^ However, the quality of evidence if these systematic reviews was low because of a serious risk of bias (i.e., the risk of a systematic error or deviation from the truth, in results or inferences).^3^ In our study, the different screen‐based devices were not separated to perform the cluster analysis, so it was not possible to determine if different types of sedentary behaviors may be related in a different way on these variables.^3^, ^5^ Given that the intensity of physical activity may influence the relationship with physical fitness,^3^, ^5^ another possible explanation could be because the instrument used to measure physical activity in this study did not allow us to know its intensity, so it was not possible to know whether the adolescents of the different profiles participated in physical activity at a higher or lower intensity. In addition, the profile with low screen time on school and non‐school days could also have high non‐screen‐based sedentary time (sedentary behaviors related to social time or educational time). Therefore, more studies analyzing not only the time spent on different screen‐based devices during school and non‐school days, but also non‐screen‐based sedentary time, and the intensity of physical activity, are needed to shed light on the relationship between these variables.

Finally, significant differences between the four identified profiles and academic performance were found. Our results indicate that adolescents who spent a high screen time during school days reported poorer academic performance, while high screen time on non‐school days does not appear to be detrimental if accompanied by low screen time on school days. The majority of previous studies also showed an inverse association between screen time and academic performance.^18^, ^61^, ^62^, ^63^, ^64^ Our results are consistent with previous research that showed that the screen time spent by adolescents during school days is more damaging than on non‐school days on academic performance.^12^, ^13^, ^14^ In fact, some studies have found a positive relationship between a high amount of screen time on non‐school days and academic performance.^12^, ^13^, ^14^ These more detrimental outcomes could also be explained by the displacement hypothesis,^17^ which suggests that excessive screen time on school days could interfere with academic activities such as doing homework, studying, etc. Previous research also suggested that recreational screen time could replace other activities such as physical activity or sleep duration, due to the finite amount of time in a single 24‐h period,^18^ leading to lower academic performance. However, the results of this study suggest that a high amount of screen time on non‐school days may not necessarily interfere with academic performance if adolescents also spend time on academic activities.

### 4.1 Limitations and future directions

The present study has some limitations that provide future research avenues. First, its cross‐sectional design precludes drawing conclusions about the cause and effect of screen time profiles and health and educational indicators. Therefore, further longitudinal studies are needed to confirm these screen time profiles, the direction of the relationships examined, and how these profiles differ in terms of health and educational indicators. Second, although the instrument used to measure screen time is valid and reliable, it did not allow us to know the purpose and context in which it was used and whether it was used actively or passively.^65^ There is a need to develop new valid and reliable instruments to measure screen time on different screen‐based devices, differentiating them by mode (active or sedentary), purpose (recreational or educational), and context of use (school or leisure time). Third, although the questionnaires used to assess physical activity and sleep duration were valid and reliable, they were self‐reported. Future studies should use device‐measured physical activity and sleep duration. Fourth, because total screen time on school and non‐school days was examined, it was not possible to determine whether some screen‐based devices might have been more harmful than others. Future studies should determine whether time spent on school and non‐school days on each screen‐based device (e.g., TV, video games, computer, smartphone, tablet, etc.) could be more or less detrimental to different health and educational indicators. Finally, given that the study was conducted in a non‐representative sample of Spanish adolescents, the results cannot be generalized to all adolescents or other age groups. Future studies should be conducted with a representative sample of Spanish adolescents from different regions.

### 4.2 Strengths

Despite the limitations noted above, this research presents several strengths that should be highlighted. First, the relatively large sample of adolescents that participated in this study ensured that there was a sufficient sample in each profile regarding statistical power. Second, this is the first study that identified school and non‐school screen time profiles using a person‐centered approach. Third, few studies have examined the possible differences between screen time on school and/or non‐school days and a wide range of health and educational indicators and none using a person‐centered approach. Finally, unlike most previous studies, four of the most used screen‐based behaviors have been examined.

## 5 CONCLUSION

In conclusion, our results indicate that adolescents who accumulated a large amount of screen time on school and non‐school days reported worse health‐related behaviors and academic performance. Adolescents who had high screen time on school days reported only a short sleep duration on school days and worse academic performance than on non‐school days. Given the high values of screen time found in the four screen time profiles and their differences found in different health and educational indicators, conducting interventions to reduce screen time in these profiles becomes essential to improving adolescents' healthy lifestyles and academic performance. It seems especially important to reduce screen time on school days in such screen time profiles to improve sleep duration on school days and academic performance.

## FUNDING INFORMATION

This study has been funded by the European Community and the Ministry of Economy of Extremadura (IB16193). We gratefully acknowledge the financial support of the Ministry of Economy and Infrastructures and European Community. M.A.T‐S is supported by the Junta de Extremadura (PD18015) and European Social Fund (FSE). In addition, this research has been funded by the FEDER, the FSE and the Junta de Extremadura, with grant numbers GR21124.

## CONFLICT OF INTEREST

The authors declare that there are no conflicts existing for any authors.

## Supporting information


File S1

File S2

## Data Availability

The datasets used and/or analyzed during the current study are available from the corresponding author on reasonable request.
