# Supplementary material for: School and non‐school day screen time profiles and their differences in health and educational indicators in adolescents
Source: Scand J Med Sci Sports. 2022 Jul 27;32(11):1668–81. doi: 10.1111/sms.14214 (PMC9796428; doi:10.1111/sms.14214)
Supplement: Supplementary file 1 — File S1 [file SMS-32-1668-s002.docx]

**Supplementary file 1.** Bivariate correlations between the different study variables.

| Measurements | Correlations | | | | | | | | | |
| --- | --- | --- | --- | --- | --- | --- | --- | --- | --- | --- |
|  | 1 | 2 | 3 | 4 | 5 | 6 | 7 | 8 | 9 | 10 |
| 1. Weekday screen time (min/day) | - | 0.51^**^ | -0.11^**^ | -0.31^**^ | -0.12^**^ | -0.24^**^ | 0.02 | -0.07^**^ | 0.09^**^ | -0.22^**^ |
| 2. Weekend screen time (min/day) |  | - | -0.08^**^ | -0.09^**^ | -0.31^**^ | -0.17^**^ | 0.00 | 0.02 | 0.07^*^ | -0.10^**^ |
| 3. Physical activity (1 – 5) |  |  | - | 0.11^**^ | -0.03 | 0.27^**^ | -0.11^**^ | 0.31^**^ | 0.16^**^ | 0.15^**^ |
| 4. Weekday sleep duration (h/day) |  |  |  | - | 0.21^**^ | 0.19^**^ | -0.15 | 0.11^**^ | -0.02 | 0.22^**^ |
| 5. Weekend sleep duration (h/day) |  |  |  |  | - | 0.00 | -0.04 | -0.02 | -0.01 | 0.08^**^ |
| 6. Mediterranean diet (-4 – 12) |  |  |  |  |  | - | 0.00 | 0.11^**^ | -0.00 | 0.30^**^ |
| 7. Body fat (%) |  |  |  |  |  |  | - | -0.31^**^ | -0.44^**^ | -0.16^**^ |
| 8. Cardiorespiratory fitness (ml/kg/min) |  |  |  |  |  |  |  | - | -0.36^**^ | 0.16^**^ |
| 9. Muscular strength (z-score) |  |  |  |  |  |  |  |  | - | -0.07^*^ |
| 10. Academic performance (1 – 10) |  |  |  |  |  |  |  |  |  | - |

*Note*. ^*^*p* < 0.05, ^**^*p* < 0.01.
