# Supplementary figures and images for: School and non‐school day screen time profiles and their differences in health and educational indicators in adolescents
Source: Scand J Med Sci Sports. 2022 Jul 27;32(11):1668–81. doi: 10.1111/sms.14214 (PMC9796428; doi:10.1111/sms.14214)

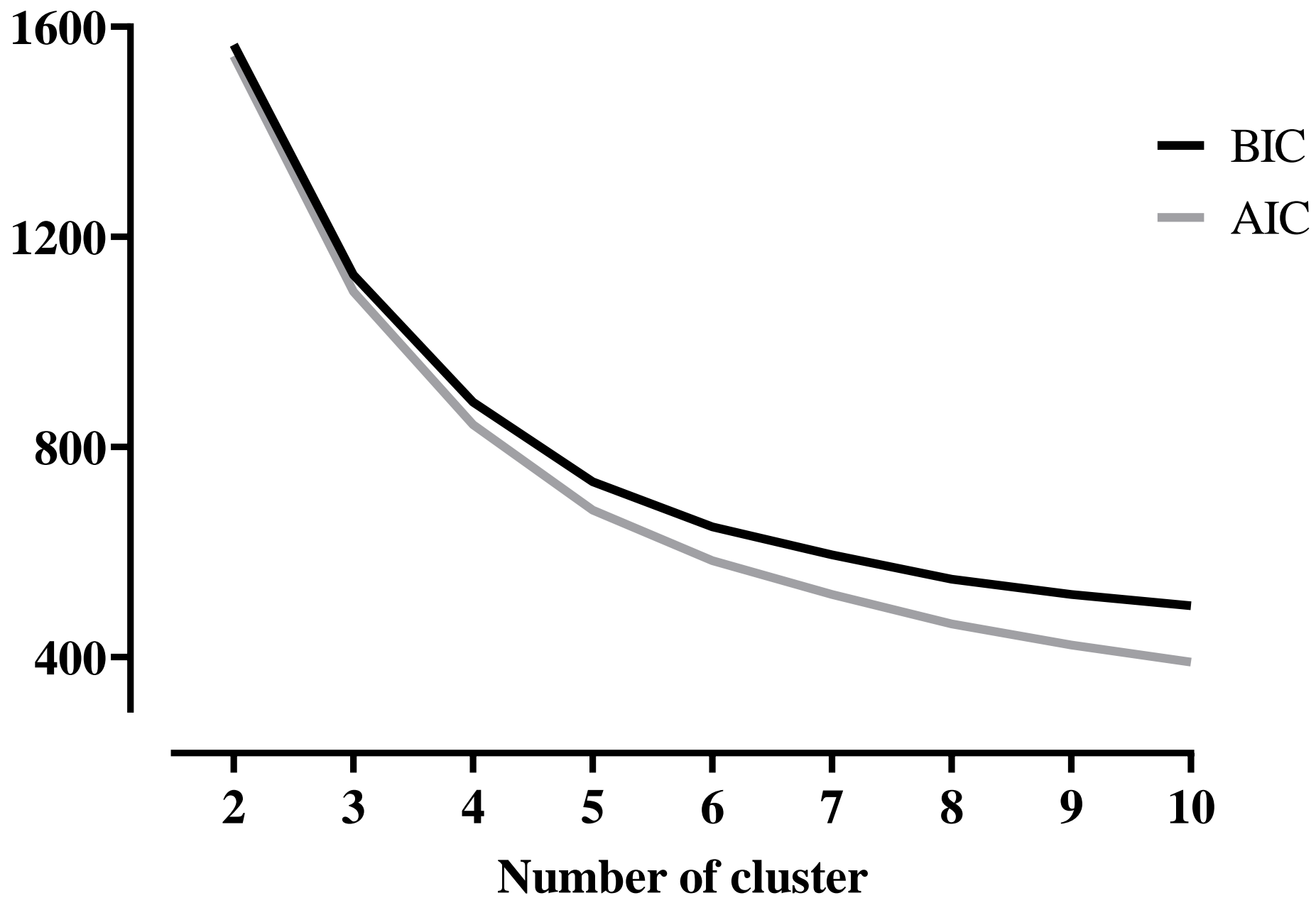

Supplement: Supplementary file 2 — File S2 [file SMS-32-1668-s001.pdf]
